# In the Tradition of Science: An Interview with Victor Ambros

**DOI:** 10.1371/journal.pgen.1000853

**Published:** 2010-03-05

**Authors:** Jane Gitschier

**Affiliations:** Department of Medicine and Pediatrics, University of California San Francisco, San Francisco, California, United States of America

I was on shaky footing with RNA interference (RNAi) and microRNAs (miRNAs), and I knew I had to do something about it. As the number of miRNAs in humans escalated and I tried to sort through the twists and turns of the compelling story of their discovery, I turned to a colleague for insight. “Interview Victor Ambros,” he said, and I took his advice.

For those of you who might also benefit from a little primer on the topic, RNAi is a well-established phenomenon of using double-stranding RNA to effect gene silencing, and it flourished as an investigative tool years before its connection to the tiny endogenous miRNAs was made. RNAi had been first recognized in plants as a response to infections, and the cellular machinery, such as Argonaut and Dicer, to effect RNAi had also emerged. But these advances had been made without appreciating the cellular fleet of stealth molecules—miRNAs—that had piloted under our radar, scanning and tempering our genome.

**Image 1:**
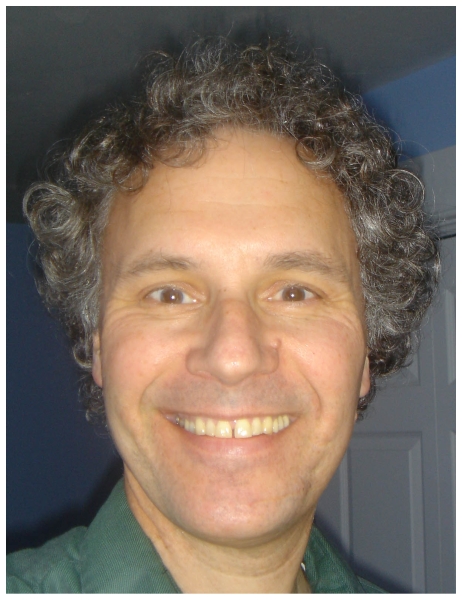
Victor Ambros.

I asked Victor Ambros to fill me in on some of these discoveries, moments he shared with his wife Rosalind and his long-term scientific collaborator and friend, Gary Ruvkun. After I had to abort plans to visit Victor in Massachusetts, we eventually settled on a Skype interview, and I persuaded him to shoot his own photo on his computer's photo booth (Image 1). We had a grainy connection but a lot of fun.

Victor grew up on a small farm in Vermont, his father and mother having made the commitment to a rural life, where they set about raising a family of eight children. He went to MIT for undergrad, grad, and post-doctoral work, ventured down Massachusetts Avenue to Harvard for his first job, and managed to slip out of state to Dartmouth for his second. He then returned to the Boston area where he has now settled in at the University of Massachusetts, Worcester.

**Gitschier:** What do you think funneled you into a career in science?

**Ambros:** I'm not sure. My earliest recollection was that I dreamed of being a baseball player. But that was until about age 8 or 9. After that, I can't recall not wanting to be a scientist, and I must trace it to reading books that were lying around the house.

I just got intrigued by the tradition of doing science. I read a book about famous inventors and books about astronomers, and decided I wanted to be an astronomer. These were plans and dreams that just sort of came together without any kind of authentic, realistic experience. Just a child reading books and deciding that's what he wanted to do. It seemed like it was a wonderful tradition to be part of—that tradition of scientists and inventors.

Somebody got me a toy telescope when I was young, and I became an amateur astronomer when I was 11 or 12. I built a telescope out of a book. My father encouraged me an awful lot. He was excited that I was interested in science and he would help me with building projects.

**Gitschier:** He was a hands-on kind of a guy.

**Ambros:** Yeah, my dad is exceedingly clever. I'd say he is a brilliant man who, because he was born at the wrong time in Europe—in Poland—was caught up in World War II. He went to high school only for a year or so because the schools closed down at the onset of the war. He became essentially a fugitive from the Russians and Germans in Poland. He was captured by the Germans and spent the rest of the War as a forced laborer. He spent from the age of 15 to 19 having no education at all.

When he was liberated by the American army, he worked for the army as an aide to some army officers, and he was exposed to a lot of books in the mansions of the ex-German rich folk, which were being used by the American army as headquarters. That's how he began to teach himself English.

By the time I was born, in 1953, I came to know my dad as someone who was very, very clever, could build almost anything, and was very well-read. It was fun to listen to him talk about books that he had read, and even today we recommend books to each other and discuss them. He speaks four or five languages. My dad is someone whom I admire enormously, especially because I felt that he was someone who had missed an opportunity to be a formally educated person, but he still made a great life for himself and his family.

I remember from a very young age being very conscious of pleasing my dad because of the contrast between what I felt I had, which were all sorts of opportunities, and the opportunities that he missed. So that would help keep me on track—study hard, because after all, that'll please Dad. So he was a very important person for me throughout my childhood and high school. He still is!

**Gitschier:** Do you mind if I just follow up a little bit more? When you said he was in a forced labor camp, do you mean he was in one of the concentration camps?

**Ambros:** He's not Jewish; he was Catholic, so he was lucky enough not to be categorically sent to death. He was also able-bodied, so he became incorporated into this system of forced labor that they had in Germany. It was very much like American slavery. People were property. They were essentially rented out or leased to others who were doing work for the government. So my dad became property of the German government, and he worked for a company that processed wood into fuel for trucks.

**Gitschier:** What happened to his siblings and his parents?

**Ambros:** Well, his mom and dad had already died when he was still a child. But he had a sister. He lost track of her during the war, but they were reunited in 1960. The Red Cross had a system of registering displaced persons. Eventually names were matched up but it took some years—this was pre-computers. They did not know that the other had survived the war.

She came over here and lived near us until she died just last year.

**Gitschier:** Do you think you've stayed in New England all these years because of the proximity to your family?

**Ambros:** Yeah, I would say so. I like New England, and it is nice to be within striking distance. But I did go to MIT, not because it is in New England, but because it was the place I wanted to go to school. We ended up being dug in, in Boston. Also, I'm a person who does like the familiar. Given a choice, I would stay.

**Gitschier:** Let's move on to some of these major discoveries that you've made. And let's start with your being in Horvitz's lab and working on these things called heterochronic mutants.

**Ambros:** The term heterochrony referred to a mode of developmental change in evolution, where animals would acquire some change in the relative timing of events, and that would lead to changes in morphology. The classic example is the axolotl, in which the adults retain their gills instead of going through the metamorphosis. Stephen Jay Gould had written extensively about these in his columns in the *Natural History* magazine. When Bob and I started studying the mutants that had primarily changes in the relative timing of events, we thought it would be cool to co-opt that term to describe the mutants, since the term was already there.

Bob had set up this group at MIT that was bringing a really interesting approach, I thought, to this worm, which was to isolate mutants that were defective in egg laying. And Bob's brilliant insight was that there are so many different ways that a worm could be defective in this behavior of egg laying that it allows access to all kinds of processes and pathways in the animal. A worm can fail to lay eggs because it's missing the apparatus and those would include all sorts of developmental mutants, and from that came the heterochronic mutants, which are the developmental timing mutants that lead to morphological problems in egg laying, and all the signaling mutants, the Ras pathway.

**Gitschier:** But he couldn't have known at the time that there was going to be a Ras-pathway mutant.

**Ambros:** It's hard to know what Bob actually anticipated. I think that he anticipated more than we give him credit for—whether it was Ras or FGF [fibroblast growth factor] or you name it, he knew that the animal was developing with enormous precision. Cells were talking to each other and neurons were connecting with muscles. So he got mutants in muscles, nerves, neurotransmitters, development, cell lineages, etc. So actually, these heterochronic mutants were a small subset of a whole series of different classes of mutants that were coming out of those egg-laying screens.

So he assigned the project to me to look at the first of these, which was *lin-4*, and another gene called *unc-86*. But I didn't really get any traction with *unc-86*.

*lin-4* was the gene that I actually made some progress on, and that was because suppressors of *lin-4* arose spontaneously. One of the first was isolated by Chip Ferguson, who was in the lab at the time. Chip gave this mutant to me and said this mutant suppresses *lin-4*, and it turned out to be a mutation in *lin-14*.

That made the link between *lin-4* and *lin-14*, and my contribution was to find some dominant mutations in *lin-14* that had the same phenotype as *lin-4*. So, the loss of function in *lin-4* was equivalent in phenotype to a gain-of-function mutation in *lin-14*. And then we did some epistasis work and decided that a parsimonious scenario was that *lin-4* repressed *lin-14*.

Then Gary Ruvkun came to Bob's lab. He was a molecular biologist, and nobody in the lab was doing molecular biology. So Gary taught us how to make DNA and do restriction digests. Gary and I collaborated on trying to clone *lin-14*. We made some progress, and we eventually published a paper showing that we had cloned *lin-14*, without including a sequence! In those days you could get a publication by demonstrating that you had identified a band on a Southern and a piece of cloned probe that represented the gene.

Then Gary focused on the *lin-14* project in his lab at MGH [Massachusetts General Hospital] and I and I took the *lin-4* project to my lab at Harvard.

**Gitschier:** I know that you and Gary are very close. Was it part of the design that you were going to stay physically close together in the Boston area?

**Ambros:** No, that was just accidental. And splitting up the genes was a good idea. In those days, cloning a gene wasn't that straightforward. You didn't have a genome sequence. We were cloning genes purely based on mutation. Transformation rescue hadn't been established yet. Each of us started in our labs in '84…

**Gitschier:** It was almost a decade then before….[you published on *lin-4*]…

**Ambros:** [Laughter] Yeah. Well, we had lots of other projects. What was done in my lab was driven by the interests of the students and the post-docs.

Also, *lin-4* was a tough project because there was only one mutation. Even though Bob had been screening and screening for egg-laying defective mutants, and *lin-4* was an egg-laying defective mutant, there was only one allele!

**Gitschier:** In retrospect, do you think that told you that it was going to be a really small gene?

**Ambros:** Well, we had lots of concerns. There were categories of concerns. One would be that it was a peculiar mutation, called E912. E means it was identified in England in [Sydney] Brenner's lab, it was actually induced by ^32^P degeneration. John Sulston had been making ^32^P-labeled worms for doing Cot curves, and a member of the lab screened the progeny of those animals for mutations and ended up getting E912.

So, we were concerned that maybe it's a peculiar kind of [DNA] rearrangement that fuses this thing to that thing in some way and has what's called “neomorphic” activity. So you might be cloning a locus that in retrospect might not really tell you anything about the normal function of either of the respective genes.

**Gitschier:** Did that kind of concern make it a back burner project?

**Ambros:** Exactly. To clone these genes, we like to proceed by getting multiple mutations, so that when we get to the gene, we'll be able to identify these mutations.

It really wasn't until my wife, Rosalind Lee, joined the lab, which I think was in 1987, that this really seemed like a perfect project for a research assistant. She was a technician and her career didn't depend on this. She came to try to move along the genetic experiments we were doing to find the locus.

And then Rhonda Feinbaum joined the lab as a post-doc. Rhonda was interested in the project, especially as this would be a team effort between her and Rosalind; it wouldn't be all on one person's shoulders. And over the course of 4 years, the two of them ended up, by dividing up the effort, putting together that story which we ended up publishing in 1993.

Rosalind [known to her friends and colleagues as Candy] did the positional cloning, mapping *lin-4* with respect to recombinant chromosomes between two worm strains, and eventually found the DNA lesion associated with the *lin-4* mutation using Southern blotting. And it turned out it, the lesion proved to be a deletion and rearrangement, consistent with a ^32^P degeneration.

Rhonda did complementation rescue experiments, and she and Candy together made the constructs and whittled the gene down. At some point we recognized that the gene product couldn't be a conventional protein-coding gene because we narrowed it down to what looked like an intron of a protein-coding gene. We were able to get rescue of the mutant phenotype fully with just the little piece of DNA—about 700 base pairs.

In parallel, Rhonda also did a very massive screen for new alleles and picked up one new allele, and that turned out to be really useful. This was a point mutation affecting a single nucleotide of the miRNA. It was the lynchpin supporting the conclusion that that the small RNA was the lin-4 gene product.

**Gitschier:** By the time you figured this all out, it was what, 1991 or '92?

**Ambros:** Yes, more or less. We wrote this down somewhere! We knew the basic story for almost a year before we published. There was a year or so where our lab and Gary Ruvkun's lab were cleaning up loose ends and putting together a nice pair of complementary papers.

The importance of that relationship with Gary's lab was that Gary was pursuing an analysis of gain-of-function mutations in *lin-14*. Those dominant mutations that were causing *lin-14* to be essentially constitutively expressed developmentally and de-repressed from *lin-4* activity were in the 3′ UTR.

Gary had cloned a gene from another nematode species and did RNA alignments to try to identify conserved sequences that may be important. He was developing a hypothesis that the 3′ UTR was a site where the *lin-4* gene product would bind. So it became really important to find out what the *lin-4* gene product was.

We anticipated that the stories would converge, so we were staying in touch. When Rhonda and Rosalind were zeroing in on this small piece of DNA and showing ultimately that the transcript from that region was really short—the main transcript was only 21 or 22 nucleotides. At that point we shared the sequence with Gary because he said, “We have sequences from these two species, and we should line them up and see whether there is some sort of anti-sense base pairing.”

It was actually pretty obvious once we did the alignment that there had to be anti-sense base pairing. *lin-4* matched the *lin-14* 3′ UTR in several places, and all of those places were conserved between the two species. And Candy had shown that *lin-4* was conserved between those species, as well, so here we had a little, well-conserved RNA, and the complementarity to *lin-14* was conserved. That was really cool.

**Gitschier:** Were you feeling pressure from Gary to get this *lin-4*?

**Ambros:** I'm sure there was a healthy competitive component there. I was thinking, “Well this matters to somebody else, so we really need to push it forward.”

**Gitschier:** Sounds more collaborative, though, than competitive.

**Ambros:** Well, from my perspective the competitive aspect was [that] you wanted to do at least a good a job as Gary was doing in his part. The pressure was: you don't want to get your part wrong! It was very nice that the sharing of the data and looking at the RNA sequence together came from a desire to make sure that the experience was a good one—good for me and for him. I didn't want to be the one who missed it when he got it. And I didn't want to be the one who got it if he missed it, because that wouldn't feel right either. So we said, we'll exchange sequences and we'll look at it together.

**Gitschier:** Were you on the phone while you were both looking at it?

**Ambros:** We sent the sequences to each other and said let's look at this and call back this evening and see what we see. So we called and said, “Do you see it?” “Yes, I see it!”

**Gitschier:** That must have been really exciting.

**Ambros:** It was. And it felt good. That we had found something and that we had found it together.

**Gitschier:** OK, now let's talk briefly about RNAi and how you eventually realized that *lin-4* fitted into that story.

**Ambros:** The phenomenon of RNAi had been described and studied in plants before Fire and Mello had hit on this double-stranded RNA [dsRNA] trigger concept in 1999. So it was already known that this phenomenon in plants and animals seemed to smell the same—an epigenetic gene silencing mechanism.

That didn't immediately help us appreciate miRNAs. We had found *lin-4* in 1993, and even though we showed it formed a dsRNA precursor, we didn't connect that to dsRNA-based phenomenon called RNAi that Fire and Mello found.

**Gitschier:** Because at that time, the RNAi people were talking about things that were brought in to the cell.

**Ambros:** That's right. At that point, it was a mysterious capacity for the animal to respond. So it wasn't clear what this represented in terms of endogenous mechanisms. The animal was too good at it for it not to be deeply important. And then there were a rapid series of discoveries, where Mello, in one of his important contributions that helped win the [Nobel] Prize for him, was finding Argonaut—that there were these conserved proteins that were required for the silencing in worms.

But, it wasn't until David Baulcombe found that the silencing process in plants involved the formation of very short—about 22-25 nucleotides—dsRNAs, that indicated that in plants, and probably by implication in animals, that the long double-stranded RNA precursor was being processed to a short molecule.

And I remember seeing that result and thinking, “Hmm, that looks a lot like *lin-4*.”

The point of the story I'm going to tell you now is how interesting it is that we—at least I—was so resistant to a new idea. We thought that *lin-4* could be just specific to worms, because Candy had actually tried to find *lin-4* molecules in other species and couldn't find them. And now we know that this little RNA isn't conserved well enough to detect by hybridization.

So, Baulcombe has found this stuff in plants and it's associated with RNAi. So maybe this helps explain how *lin-4* biogenesis works; it must be that it has co-opted the RNAi machinery to be processed. So, you see what I'm saying—not that *lin-4* represented some broad class of things…

**Gitschier:**… that were fundamental.

**Ambros:** Yeah. Maybe it's just a special case of how a system can co-opt the RNAi machinery to make a gene product that is a small RNA product.

And it wasn't until finally Gary found *let-7* in *C. elegans*, with a sequence completely different from *lin-4*, that [we realized] in nematodes the same thing had evolved twice. But personally, it didn't trigger me to think that *lin-4* and *let-7* must be part of something very broad and conserved in all animals. Still, in my mind it was a special nematode thing.

Until Gary published his *Nature* paper in 2000 showing that *let-7* was conserved in sequence in all these animals—sea urchins, mammals… And that was a total revelation. It was a watershed discovery that made me instantly go from a pessimist to an optimist. I said to myself that there must be more small RNAs like *lin-4* and *let-7*, and in other animals. It was really exciting. To open that *Nature* and say, “Holy cow! The way I've been looking at things is totally wrong.”

**Gitschier:** Didn't you know about Gary's work before the paper came out?

**Ambros:** He kept it secret! It was really cool.

**Gitschier:** I loved what you said in the Lasker Award commentary [in *Nature Medicine*] that after reading their paper you literally had to sit down for 10 minutes and look out the window to reorder your view of the universe.

**Ambros:** Right, so Candy and I immediately started cloning those things, and we were very naïve; we thought we were the only ones doing it. That was another adventure.

**Gitschier:** Actually, that was another thing I liked about both your and Gary's commentaries. It was just so great the way you referred to your spouses as being important contributors to the work. I was touched.

**Ambros:** I think that what Gary and I were trying to get at, independently, was the question of what's the point, really, of an award, like the Lasker Award? Basically, I feel lucky to be there, because if it weren't for a whole lot of stuff that I did not control, the award wouldn't have happened. If I hadn't happened to work in David Baltimore's lab, I probably wouldn't have been noticed by Bob Horvitz, and if I hadn't been in Bob Horvitz's lab, I wouldn't have even worked on this system. And, if Candy hadn't come to work in the lab, none of this project would have happened. And, if my father hadn't encouraged me… It just gets out of control if you think about what can lead to a moment like getting an award.

And so it has very little to do, frankly, with the particular person getting the award. What the award represents is a process that involves interactions amongst many, many people. And the end, one person ends up getting the award. It's really important to try to acknowledge that and understand the fact that really everything that happens in science, including the discoveries that people try to acknowledge by awards, are really the products of this confluence of people's histories and people's interactions. I really believe that science gets done by people with average abilities and talents, for the most part, and when something special happens, enough so that people want to acknowledge it with an award, it was really…in large part…luck!

We try to say to the public, here's an award for somebody who's really, really special. But actually, it's not the *somebody* who is really special, it's the *science* that is special. The way we do science, and the way it works is so amazing. I wish non-scientists would better understand this. That science is a community exercise, that it involves people interacting, that it involves a lot of good fortune in the context of people trying to do something really carefully and following curiosity. That's why it works so well!

You're preaching to the choir, but the idea is that science remains fun and it is a tremendous adventure. It's great that you do these columns because it reminds people of why we all do science. It's through the stories that we are reminded.

